# Publisher Correction: Adaptation of the small intestine to microbial enteropathogens in Zambian children with stunting

**DOI:** 10.1038/s41564-021-01042-3

**Published:** 2021-12-08

**Authors:** Beatrice Amadi, Kanekwa Zyambo, Kanta Chandwe, Ellen Besa, Chola Mulenga, Simutanyi Mwakamui, Stepfanie Siyumbwa, Sophie Croft, Rose Banda, Miyoba Chipunza, Kapula Chifunda, Lydia Kazhila, Kelley VanBuskirk, Paul Kelly

**Affiliations:** 1 Tropical Gastroenterology and Nutrition Group, University of Zambia School of Medicine, Lusaka, Zambia; 2 Blizard Institute, Barts and The London School of Medicine and Dentistry, Queen Mary University of London, London, UK

**Keywords:** Predictive markers, Intestinal diseases, Infection, Molecular medicine

Correction to: *Nature Microbiology* 10.1038/s41564-020-00849-w, published online 15 February 2021.

In the version of this article initially published, there was a labeling error in Fig. 3a. Specifically, the second (mauve) term in the color key, now reading “Enterotoxigenic *Escherichia coli* (ETEC),” initially appeared as “Shiga toxin expressing *E. coli*.” The change has been made to the HTML and PDF versions of the article.

