# Supplementary material for: Adaptation of the small intestine to microbial enteropathogens in Zambian children with stunting
Source: Nat Microbiol. 2021 Feb 15;6(4):445–54. doi: 10.1038/s41564-020-00849-w (PMC8007472; doi:10.1038/s41564-020-00849-w)
Supplement: Supplementary file 1 — Supplementary Tables 1–6. [file 41564_2020_849_MOESM1_ESM.pdf]

nature portfolio

https://doi.org/10.1038/s41564-020-00849-w

Supplementary information

Adaptation of the small intestine to microbial enteropathogens in Zambian children with stunting

In the format provided by the authors and unedited

SUPPLEMENTARY TABLES

Supplementary Table 1 Pathogen detection in cases and controls at baseline

	Stunted children	Controls	*P*
Rotavirus A	62 (23)	1 (4)	0.03
Norovirus	158 (60)	6 (26)	0.002
Adenovirus	34 (13)	2 (9)	0.75
Salmonella spp	199 (75)	11 (48)	0.007
Shigella spp	176 (67)	5 (22)	0.0000
Campylobacter spp	135 (51)	5 (22)	0.008
Enterotoxigenic *Escherichia coli* (ETEC)	183 (69)	5 (22)	0.0000
Stx-expressing *Escherichia coli* (STEC)	21 (8)	0	0.39
Escherichia coli O157	12 (5)	1 (4)	1.00
Clostridium difficile	12 (5)	0	0.61
Cryptosporidium spp.	74 (28)	4 (18)	0.34
Giardia intestinalis	186 (71)	11 (48)	0.03
Number of pathogens detected in each stool sample (median, IQR)	5 (3-6)	1 (0-3)	0.0001 by Kruskal-Wallis test

Figures shown are n(%). No cases of *Yersinia enterocolitica* were detected; two infections with *Vibrio cholerae* and one of *Entamoeba histolytica* were detected and are therefore not shown. The xTAG Gastropanel does not include EPEC or EAggEC primers. Hypothesis testing for each pathogen used a 2-sided Fisher's exact test, but the difference in number of pathogens used the Kruskal-Wallis test.

Supplementary Table 2 Regression analysis of effect of age and control status on pathogen burden, restricted to first sample collected (n=195)

**Pathogen**	**Age**		**Control status**<br>181 cases, 14 controls	
**Linear regression of total pathogen number**				
	β	***P***	β	***P***
Total pathogen number	0.10<br>(95%CI 0.02,0.18)	0.04	-2.25<br>(95%CI -3.8,-1.1)	0.001
**Logistic regression of individual pathogens**				
	Odds Ratio (95%CI)	***P***	Odds Ratio (95%CI)	***P***
ETEC	1.08 (1.008,1.17)	0.03	0.18 (0.04.0.69)	0.01
Salmonella spp.	0.92 (0.84, 1.01)	0.07	0.10 (0.03,0.38)	0.001
Shigella spp.	1.08 (1.003,1.16)	0.04	0.20 (0.05,0.80)	0.02
Campylobacter spp.	1.02 (0.95,1.09)	0.59	0.09 (0.01,0.74)	0.03
Norovirus	0.99 (0.93,1.06)	0.83	0.16 (0.04,0.64)	0.009
Rotavirus	1.05 (0.97,1.14)	0.23	-	-
Cryptosporidium spp.	1.06 (0.98,1.15)	0.12	0.69 (0.14,3.48)	0.65
Giardia intestinalis	1.17 (1.08,1.27)	<0.001	0.88 (0.27,2.87)	0.83

Output of linear and logistic regression models using pathogen number and pathogen positivity as the dependent variable, and age and control status as independent variables. No OR could be obtained for rotavirus, as none of the controls carried rotavirus (*P*=0.04 by 2-sided Fisher's exact test).

Supplementary Table 3 Brush border gene expression levels and correlations with age and translocation markers in children with non-responsive stunting

Gene			Correlation (ρ)				
Symbol	Name/function	Mean expression level (FPKM)	Age	LPS	LBP	sCD14	iFABP
ANPEP	Alanyl aminopeptidase	2093					
ALPI	Alkaline phosphatase	1279					
LCT	Lactase	333					
PRSS2	Anionic trypsinogen	267					
TMPRSS15	Enteropeptidase	250					
FOLH1	Glutamate carboxypeptidase II (GP2), folate hydrolase	203		ρ = -0.37 (*P*=0.049)			
ACE	Angiotensin 1 converting enzyme	180		ρ = -0.45 (*P*=0.03)			
GGT1	γ-glutamyl transferase	175					
TREH	Trehalase	174					
SI	Sucrase-isomaltase	165					
PRSS3	Mesotrypsinogen	139					
MGAM	Maltase-glucoamylase	104		ρ = -0.46 (*P*=0.01)			
DPP4	Dipeptidylpeptidase IV	70					

Correlation coefficients shown are Spearman's coefficients, and P values were not adjusted for multiple comparisons (see Online Methods). Blank cells indicate that no significant correlation was found (*P* > 0.05). For age, n=30, but for biomarkers n=29.The effect of ACE on reducing circulating LPS might be related to observed effects of ACE2 on LPS-induced inflammation: Ye R, Liu Z. ACE2 exhibits protective effects against LPS-induced acute lung injury in mice by inhibiting the LPS-TLR4 pathway. *Exp Mol Pathol*. 2020;113:104350. doi:10.1016/j.yexmp.2019.104350. LPS, lipopolysaccharide; LBP, LPS binding protein; sCD14, soluble CD14; iFABP, intestinal type fatty acid binding protein.

Supplementary Table 4 Correlations between biomarkers of microbial translocation, age, and expression of selected genes known to contribute to barrier function in children with non-responsive stunting

Gene			Correlation (ρ)				
Symbol	Name/function	Mean expression level	Age	LPS	LBP	sCD14	iFABP
MUC1	Mucin 1	17.4					
MUC2	Mucin 2	8.5					
MUC4	Mucin 4	1.6			ρ = -0.45 *P* = 0.01		
MUC6	Mucin 6	16.4					ρ = -0.38 *P* = 0.04
MUC13	Mucin 13	954		ρ = -0.38 *P* = 0.04			
MUC17	Mucin 17	52		ρ = -0.37 *P* = 0.048			
MUC20	Mucin 20	11.6					
SLC9A3	NHE3 – bicarbonate transporter	65					
TFF1	Trefoil factor 1	51					
TFF2	Trefoil factor 2	59					ρ = -0.44

							P = 0.02
TFF3	Trefoil factor 3	97					
SPDEF	SAM pointed domain containing ETS transcription factor	3.3					
AGR2	Anterior Gradient 2, protein disulphide isomerase family member	144					
CLCA1	Chloride channel accessory 1	54					
ZG16	Zymogen granule protein 16	59					
FCGBP	Fc Fragment Of IgG Binding Protein	14					
DEFA5	Defensin alpha 5	2387					
DEFA6	Defensin alpha 6	1080					
CAMP	Cathelicidin antimicrobial peptide (LL-37)	1.1					ρ = -0.50 *P* = 0.006
DEFB1	Defensin beta 1	7					
CLDN2	Claudin 2	41					
CLDN 3	Claudin 3	161					
CLDN 4	Claudin 4	286		ρ = -0.42 *P* = 0.01		ρ = -0.46 *P* = 0.01	
CLDN 5	Claudin 5	13					
CLDN 7	Claudin 7	451		ρ = -0.45			

				P = 0.03			
CLDN 12	Claudin 12	7					ρ = -0.50 *P* = 0.006
CLDN 15	Claudin 15	288					
CLDN 23	Claudin 23	35					
OCLN	Occludin	11					
TJP1	Tight junction protein 1 (ZO-1)	8					
TJP2	Tight junction protein 2 (ZO-2)	31					
TJP3	Tight junction protein 2 (ZO-3)	68					
CGN	Cingulin	64					
CTNNB1	β- catenin	89					

Correlation coefficients shown are Spearman's coefficients, and P values were not adjusted for multiple comparisons (see Online Methods). Blank cells indicate that no significant correlation was found (*P* > 0.05). For age, n=30, but for biomarkers n=29. Related genes with expression levels below 1 were not included; this included MUC5B and all β-defensin genes except DEFB1. None of these genes varied in expression with age when the biopsy was taken. Blank cells indicate that no significant correlation was found (*P* < 0.05). LPS, lipopolysaccharide; LBP, LPS binding protein; sCD14, soluble CD14; iFABP, intestinal type fatty acid binding protein.

Supplementary Table 5 Correlations between biomarkers of microbial translocation, age, and expression of selected nutrient transporter genes in children with non-responsive stunting

Gene			Correlation (ρ)						
Symbol	Name/function	Mean expression level (FPKM)	Age	LPS	LBP	sCD14	iFABP	VH	CD
SLC2A5	GLUT-5 (fructose)	170						ρ = 0.44 *P* = 0.03	
SLC5A1	SGLT1 (glucose)	412							
SLC3A1	Amino acids	69		ρ = -0.43 *P* = 0.02					
SLC3A2	Amino acids	122							
SLC7A7	Amino acids	99							
SLC7A9	Amino acids	92							
SLC6A19	Amino acids	217							
SLC15A1	PEPT1 (dipeptides)	193							
SLC9A3	NHE-3 (bicarbonate)	65							
SLC26A3	DRA (chloride)	182							
SLC13A2	Dicarboxylic acids	157							
SLC22A18	Organic cations	130			ρ = 0.47 *P* = 0.01				

SLC20A2	Phosphate	39								
SLC27A4	Fatty acids									
SLC28A2	Nucleosides (purine)	91								
SLC11A2	DMT-1 (divalent cations)	156								
SLC39A4	ZIP4 (zinc)	331								
SLC40A1	Ferroportin (iron)	303								
SLC19A1	Folate	42								
SLC19A2	Folate	1.3								ρ = -0.45<br>*P* = 0.02
SLC46A1	Folate	146								
SLC52A1	Riboflavin	92								

Correlation coefficients shown are Spearman's coefficients, and P values were not adjusted for multiple comparisons (see Online Methods). Blank cells indicate that no significant correlation was found ($P > 0.05$). For age, n=30, but for biomarkers n=29. LPS, lipopolysaccharide; LBP, LPS binding protein; sCD14, soluble CD14; iFABP, intestinal type fatty acid binding protein; VH, villus height; CD, crypt depth.

Supplementary Table 6 Impact of HIV status on biomarkers and anthropometry, in stunted children only, at baseline

	**HIV infected (n=10)**	**HIV exposed but uninfected (n=83)**	**HIV unexposed (n=198)**	***P***
Anthropometry: LAZ	-2.82 (-3.3, -2.4)	-2.74 (-3.2, -2.4)	-2.51 (-3.1, -2.1)	0.02
Anthropometry: WLZ	-1.62 (-2.9, -0.8)	-1.46 (-1.8, -0.7)	-1.25 (-2.0, -0.6)	0.26
LPS (EU/ml)	378 (205, 934)	462 (280, 674) n=80	388 (259, 585) n=189	0.59
LBP (ng/ml)	26.8 (11.1, 50.0)	14.1 (9.8, 22.3) n=80	15.9 (10.7, 25.6) n=195	0.12
sCD14 (mg/l)	2.9 (2.0, 5.3)	3.1 (1.8, 4.4)	3.3 (1.9, 4.6) n=197	0.91
iFABP (ng/ml)	0.397 (0, 0.842) n=8	0.937 (0. 374, 1.612) n=77	0.838 (0.309, 1.670) n=171	0.09
GLP2 (ng/ml)	2.7 (2.1, 4.2)	3.8 (2.3, 5.4)	2.9 (1.7, 4.4)	0.14

Values shown are median and interquartile range (IQR); LAZ, length-for-age *z* score; WLZ, weight-for-length *z* score. Numbers of children with data available are shown in each cell where less than the total shown at the head of the column. *P* values were obtained using the Kruskal-Wallis test.
